# Analysis of an Antibiotic Stewardship Program for Asymptomatic Bacteriuria in the Veterans Affairs Health Care System

**DOI:** 10.1001/jamanetworkopen.2022.22530

**Published:** 2022-07-25

**Authors:** Larissa Grigoryan, Aanand D. Naik, Paola Lichtenberger, Christopher J. Graber, Payal K. Patel, Dimitri M. Drekonja, Timothy P. Gauthier, Bhavarth Shukla, Anne E. Sales, Sarah L. Krein, John N. Van, Laura M. Dillon, Sylvia J. Hysong, Jennifer R. Kramer, Annette Walder, David Ramsey, Barbara W. Trautner

**Affiliations:** 1 Department of Family and Community Medicine, Baylor College of Medicine, Houston, Texas; 2 Center for Innovations in Quality, Effectiveness, and Safety, Michael E. DeBakey Veterans Affairs (VA) Medical Center, Houston, Texas; 3 Department of Management, Policy and Community Health, School of Public Health, University of Texas Health Science Center, Houston; 4 Section of Health Services Research, Department of Medicine, Baylor College of Medicine, Houston, Texas; 5 Department of Medicine, University of Miami Miller School of Medicine and the Miami VA Healthcare System, University of Miami, Miami, Florida; 6 Infectious Diseases Section, VA Greater Los Angeles Healthcare System and David Geffen School of Medicine at UCLA (University of California, Los Angeles); 7 Division of Infectious Diseases, Department of Medicine, University of Michigan and VA Ann Arbor Healthcare System, Ann Arbor; 8 Division of Infectious Diseases and International Medicine, University of Minnesota Medical School and Minneapolis VA Health Care System, Minneapolis; 9 Clinical Pharmacy Enterprise, Baptist Health South Florida, Miami, Florida; 10 Center for Clinical Management Research, VA Ann Arbor Healthcare System, Ann Arbor, Michigan; 11 Department of Learning Health Sciences, University of Michigan Medical School, Ann Arbor

## Abstract

**Importance:**

Antibiotic stewardship for asymptomatic bacteriuria (ASB) is an important quality improvement target. Understanding how to implement successful antibiotic stewardship interventions is limited.

**Objective:**

To evaluate the effectiveness of a quality improvement stewardship intervention on reducing unnecessary urine cultures and antibiotic use in patients with ASB.

**Design, Setting, and Participants:**

This interrupted time series quality improvement study was performed at the acute inpatient medical and long-term care units of 4 intervention sites and 4 comparison sites in the Veterans Affairs (VA) health care system from October 1, 2017, through April 30, 2020. Participants included the clinicians who order or collect urine cultures and who order, dispense, or administer antibiotics. Clinical outcomes were measured in all patients in a study unit during the study period. Data were analyzed from July 6, 2020, to May 24, 2021.

**Intervention:**

Case-based teaching on how to apply an evidence-based algorithm to distinguish urinary tract infection and ASB. The intervention was implemented through external facilitation by a centralized coordinating center, with a site champion at each intervention site serving as an internal facilitator.

**Main Outcomes and Measures:**

Urine culture orders and days of antibiotic therapy (DOT) and length of antibiotic therapy in days (LOT) associated with urine cultures, standardized by 1000 bed-days, were obtained from the VA’s Corporate Data Warehouse.

**Results:**

Of 11 299 patients included, 10 703 (94.7%) were men, with a mean (SD) age of 72.6 (11.8) years. The decrease in urine cultures before and after the intervention was not significant in intervention sites per segmented regression analysis (−0.04 [95% CI, −0.17 to 0.09]; *P* = .56). However, difference-in-differences analysis comparing intervention with comparison sites found a significant reduction in the number of urine cultures ordered by 3.24 urine cultures per 1000 bed-days (*P* = .003). In the segmented regression analyses, the relative percentage decrease of DOT in the postintervention period at the intervention sites was 21.7% (*P* = .007), from 46.1 (95% CI, 28.8-63.4) to 37.0 (95% CI, 22.6-51.4) per 1000 bed-days. The relative percentage decrease of LOT in the postintervention period at the intervention sites was 21.0% (*P* = .001), from 36.7 (95% CI, 23.2-50.2) to 29.6 (95% CI, 18.2-41.0) per 1000 bed-days.

**Conclusions and Relevance:**

The findings of this quality improvement study suggest that an individualized intervention for antibiotic stewardship for ASB was associated with a decrease in urine cultures and antibiotic use when implemented at multiple sites via external and internal facilitation. The electronic health record database–derived outcome measures and centralized facilitation approach are both suitable for dissemination.

## Key Points

**Question:**

Can a quality improvement antibiotic stewardship intervention implemented through external and internal facilitation decrease urine culture orders and antibiotic use for asymptomatic bacteriuria (ASB)?

**Findings:**

In this quality improvement study of 11 299 patients in acute medical and long-term care units at 4 Veterans Affairs facilities, case-based teaching on applying an evidence-based algorithm to distinguish urinary tract infection and ASB was associated with fewer urine culture orders, fewer days of antibiotics, and shorter length of antibiotic therapy. The 3 clinical outcomes did not improve at 4 comparison sites.

**Meaning:**

These findings suggest that individualized antibiotic stewardship for ASB can be implemented effectively at a distance via external and internal facilitation.

## Teaser

This quality improvement study explores external and internal facilitation as an implementation strategy for antibiotic stewardship in both acute and long-term care among patients with asymptomatic bacteriuria.

## Introduction

Antimicrobial stewardship is an important quality improvement priority.^1^ One of the important targets for stewardship is inappropriate treatment of asymptomatic bacteriuria (ASB), or bacteria in the urinary tract without related symptoms.^2^ Despite multiple evidence-based guidelines^2,3,4,5^ that recommend against culturing the urine to screen and treat ASB with antimicrobials, treatment of ASB is very common (45% in a recent meta-analysis).^6^ The US Department of Veterans Affairs (VA) Antimicrobial Stewardship Task Force reported that 72% of 1219 cases with ASB were treated unnecessarily with antibiotics.^7^

A urine culture is often the first step in a chain of events leading to unnecessary antibiotic use, longer hospital stays, and other harms.^8,9,10,11^ Therefore, investigators including members of our team^12^ designed an intervention that focuses on reducing both unnecessary urine cultures (diagnostic stewardship) and unnecessary antibiotic use (antibiotic stewardship). This intervention package, the Kicking CAUTI (catheter-associated urinary tract infection) Campaign, consists of a validated guideline-based algorithm plus individualized audit and feedback.^12^ The Less is More for ASB project was designed to evaluate whether the Kicking CAUTI intervention could be scaled up and delivered at a distance to 4 geographically distant VA medical centers with 4 comparison sites. Although we retained the Kicking CAUTI name, the intervention’s scope was expanded to address all cases of suspected UTI.

A novel aspect of the Less is More for ASB project was the exploration of external and internal facilitation as an implementation strategy for antibiotic stewardship in both acute and long-term care.^13,14,15^ Our centralized coordinating center served as the external facilitator, or the agency responsible for promoting and supporting positive change.^16^ The external facilitators (L.G., J.N.V., L.M.D., and B.W.T.) supported each intervention site’s champion, who became an internal facilitator for uptake of the intervention (P.L., C.J.G., P.K.P., D.M.D., T.P.G., and B.S.). The site champion or internal facilitator at each site was a local leader in infectious diseases, well-positioned to deliver training on how to use Kicking CAUTI components and promote organizational culture change. In the Less is More for ASB project, we hypothesized that our intervention would lead to the following outcomes: (1) the rate of urine culture orders would decrease over the course of the intervention period in intervention sites and would not decrease in the comparison sites; and (2) antimicrobial use related to urine cultures would decrease in the intervention sites and would not decrease in the comparison sites.

## Methods

### Study Design

We used an interrupted time series design with 4 intervention sites and 4 comparison sites for this quality improvement study. This project engaged 4 geographically distant VA facilities in the intervention; each was paired with a control site in the same geographic region. Our intervention and comparison sites were similar in terms of geographic location, number of beds overall, intensive care unit beds, types of wards, academic affiliation, and teaching status, but they were specifically chosen to be in different VA Service Networks to avoid cross-contamination. Standards for Quality Improvement Reporting Excellence (SQUIRE 2.0) reporting guidelines were referenced as a framework for quality improvement methods, data analysis, and reporting. Although this project was considered quality improvement from the perspective of each implementation site, the central facilitation team had access to individual health records at each site. Therefore, we sought and received approval by the institutional review board at each site. The need for informed consent was waived because the goal of the intervention was to implement standard of care based upon published guidelines.

A central coordinating center provided external facilitation, and each site champion served as an internal facilitator. External facilitation efforts included organizing monthly meetings to bring together the 4 local site teams, providing site-specific teaching cases, data compilation, and availability of 2 research coordinators (J.N.V. and L.M.D.) for advice and encouragement. The internal facilitators received standardized teaching cases that embedded the algorithm in an interactive decision tree, and they were encouraged to deliver 2 to 4 teaching cases per month in various settings (team rounds, grand rounds, in-services, etc) of their own choice.

### Intervention Overview

The intervention encompassed (1) an evidence-based algorithm that distilled the guidelines into a streamlined clinical pathway and (2) case-based education to train clinicians to use the algorithm.^17^ The Kicking CAUTI algorithm steps clinicians through 2 questions they should ask themselves before ordering a urine culture or starting antibiotics for suspected UTI (eFigure in the Supplement). The intervention targeted physicians, pharmacists, nurses, nurse practitioners, physician assistants, and clinical nurse assistants on acute and long-term care units. Each site went through 2 phases: baseline data collection and an intervention phase. During the intervention period, internal facilitators delivered interactive teaching cases to the targeted clinicians using team rounds, in-services, and teaching conferences. Cases were actual patient encounters at the intervention sites occurring within the time frame of the study. Typically, the local site champion (an infectious diseases physician and/or pharmacist) met one of the internal medicine teams in their office and reviewed one of the teaching cases from the library provided by the study, using algorithm pocket cards. The residents and students frequently asked specific questions about their own cases and received immediate advice.

**Figure 1.**
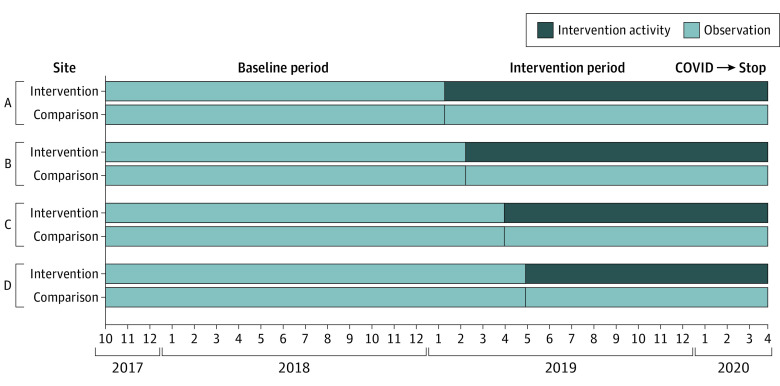
Less is More Study Timeline The 4 intervention sites entered the intervention phase at different times. For purposes of data collection and analysis, each intervention site was paired with a comparison site in the same geographic region (eg, intervention site A and comparison site A) to include 8 sites in total. Data from all sites were grouped into baseline or intervention periods.

We intended for each site to spend at least 12 months in the intervention phase. We staggered the start of the intervention across sites to focus external facilitation on 1 site at a time (Figure 1). The interruption by COVID-19 ended the intervention activities 2 months early in 1 of the 4 sites. We concluded data collection on May 1, 2020.

### Data Collection

#### Patient Cohort

We included all patients admitted to or residing in an acute care medicine ward or a community living center long-term care unit (VA nursing home) during the study period. Patient data were extracted from the VA Corporate Data Warehouse housed on the VA Informatics and Computing Infrastructure. Baseline was from October 1, 2017, until the start of the intervention in each site or its matched control; intervention was from start date through April 30, 2020 (Figure 1).

#### Measuring Antibiotic Use Temporally Associated With Urine Culture Orders

Pharmacy data on systemic antibiotic prescriptions was extracted for patients with a urine culture order. We excluded drugs not used to treat urinary organisms (eMethods 1 in the Supplement). Antibiotic prescriptions were included in the calculations for days of therapy (DOT) and length of therapy in days (LOT) if they were ordered within 1 calendar day prior or 2 days after the date the urine culture was ordered. DOT is measured by calculating the number of days a patient is prescribed each antimicrobial agent and then counting those days for each antimicrobial separately. LOT focuses on the duration of antimicrobial therapy.^18,19^ For example, if a patient is prescribed 2 antibiotics to take together for 5 days, the DOT is 10 days and the LOT is 5 days. By using both metrics, we understand the number of antibiotics used and the true lengths of therapy. Our pocket card decision algorithm (eFigure in the Supplement) had 2 sides: the front side addresses when to withhold urine cultures and antibiotics, whereas the back side addresses when to stop antibiotic therapy that proves to be unnecessary.

We used patient bed-days as a denominator to normalize urine cultures, DOT, and LOT. Bed-days were determined by linking the daily census files to the list of inpatient wards we included in our study. We excluded antibiotics reordered after a gap of longer than 1 day and prescriptions for a course longer than 30 days.

To validate that our antibiotic use measures reflected antibiotics prescribed in reaction to a suspected UTI or to the results of a urine culture, we performed a medical record review of 1261 cases of positive urine cultures in intervention sites—726 in the baseline period and 535 in the intervention period. Each culture was classified as ASB or UTI per standardized algorithm^20^ and assessed to determine whether antibiotics had been prescribed to treat the urine culture results. Of these 1261 cases, 760 (60.3%) were ASB (480 and 280 in the baseline and intervention periods, respectively). After the intervention, the percentage of ASB cases treated with antibiotics fell from 113 of 480 (23.5%) to 43 of 280 (15.3%; *P* = .01).

### Outcome Measures

The primary outcome (hypothesis 1) was the total number of urine cultures ordered by inpatient or long-term care clinicians (urine cultures ordered before admission in the emergency department or outpatient clinics were excluded). Our outcomes for antimicrobial use (hypothesis 2) were metrics of inpatient antimicrobial consumption: DOT and LOT,^18,19^ both measured for antibiotic regimens started temporally in relationship to a urine culture.

### Statistical Analysis

Data were analyzed from July 6, 2020, to May 24, 2021. The sample size calculation for this study was reported previously.^17^ We performed descriptive statistics for patients’ demographic characteristics (age, sex, race and ethnicity, and Deyo modification of the Charlson Comorbidity Index) and site of care (acute medical vs long-term care) stratified across the baseline and intervention periods and the intervention and comparison sites. Race and ethnicity are recorded in each patient’s electronic health record, and this information was included to support the generalizability of the study.

Data for each outcome variable (urine cultures and urine culture–related DOT and LOT per 1000 bed-days) were aggregated monthly by facility level, and the period was divided into baseline and intervention segments. Autoregressive integrative moving average interrupted time series techniques were used to examine change in slope or level owing to the intervention. The details of the segmented linear regression models are available in eMethods 2 in the Supplement. We also evaluated whether intervention and comparison sites had similar baseline trends in each outcome. If these trends were similar, we applied the difference-in-differences (DID) approach for that outcome. The details of the DID analysis are provided in eMethods 2 in the Supplement. Two-sided *P* < .05 indicated statistical significance. Analyses were conducted with STATA statistical software, version 17 (StataCorp LLC) and SAS statistical software, version 9.4 (SAS Institute, Inc).

## Results

Of 11 299 patients included, 10 703 (94.7%) were men, 524 (4.6%) were women, 71 (0.6%) did not disclose, and 1 (0.01%) was a transgender woman, The mean (SD) age was 72.6 (11.8) years. Demographic characteristics in the baseline and intervention periods in the intervention and comparison sites are presented in Table 1. During the baseline and intervention periods, 12 260 urine cultures were ordered in 11 299 unique patients in the acute and long-term care wards of the 8 sites over 900 437 bed-days (Table 2). Of the 12 260 urine cultures, 5867 (47.9%) were positive (Table 2). Of all 4981 treated patients, 2635 (52.9%) were prescribed at least 2 antibiotics in each study period in the intervention and comparison sites (Table 2).

**Table 1.** Characteristics of Study Patients Stratified by Study Site and Study Period^a^

Characteristic	Intervention sites		Comparison sites		Overall (n = 11 299)
	Baseline period (n = 4109)	Intervention period (n = 1696)	Baseline period (n = 3843)	Intervention period (n = 1651)	
Age, mean (SD), y	72.5 (11.9)	72.6 (12.0)	72.5 (11.8)	73.1 (11.4)	72.6 (11.8)
Sex					
Men	3903 (95.0)	1600 (94.3)	3633 (94.5)	1567 (94.9)	10 703 (94.7)
Women	187 (4.5)	88 (5.2)	182 (4.7)	67 (4.1)	524 (4.6)
Other^b^	19 (0.5)	8 (0.5)	28 (0.7)	17 (1.0)	72 (0.6)
Race					
Black or African American	839 (20.4)	382 (22.5)	602 (15.7)	287 (17.4)	2110 (18.7)
White	2905 (70.7)	1181 (69.6)	2954 (76.9)	1243 (75.3)	8283 (73.3)
Other^c^	99 (2.4)	40 (2.3)	105 (2.7)	44 (2.7)	288 (2.5)
Unknown	266 (6.5)	93 (5.5)	182 (4.7)	77 (4.7)	618 (5.5)
Ethnicity					
Hispanic or Latino	262 (6.4)	113 (6.7)	190 (4.9)	78 (4.7)	643 (5.7)
Not Hispanic or Latino	3720 (90.5)	1533 (90.4)	3536 (92.0)	1517 (91.9)	10 306 (91.2)
Unknown	127 (3.1)	50 (2.9)	117 (3.0)	56 (3.4)	353 (3.1)
Deyo comorbidity score, mean (SD)	4.7 (3.5)	4.9 (3.6)	4.7 (3.4)	4.8 (3.5)	4.7 (3.5)
Ward type					
Acute medical	3699 (90.0)	1521 (89.7)	3454 (89.9)	1461 (88.5)	10 135 (89.7)
Long-term care	410 (10.0)	175 (10.3)	389 (10.1)	190 (11.5)	1164 (10.3)

^a^ Unless otherwise indicated, data are expressed as No. (%) of patients. Percentages have been rounded and may not total 100.

^b^ Includes did not disclose and transgender.

^c^ Includes American Indian or Alaska Native, Asian, and Native Hawaiian or Other Pacific Islander.

**Table 2.** Urine Cultures Ordered for Study Patients Stratified by Study Site and Study Period

Variable	Intervention sites		Comparison sites		Overall
	Baseline period	Intervention period	Baseline period	Intervention period	
No. (%) of inpatient cultures					
All	4087	2222	3673	2278	12 260
Acute medical	3323 (81.3)	1864 (83.9)	3037 (82.7)	1912 (83.9)	10 136 (82.7)
Long-term care	764 (18.7)	358 (16.1)	636 (17.3)	366 (16.1)	2124 (17.3)
No. (%) of positive cultures					
All	2338	1226	1395	908	5867
Acute medical	1807 (77.3)	980 (79.9)	1053 (75.5)	695 (76.5)	4535 (77.3)
Long-term care	531 (22.7)	246 (20.1)	342 (24.5)	213 (23.5)	1332 (22.7)
No. (%) of bed-days					
All	270 577	187 332	261 650	180 878	900 437
Acute medical	133 263 (49.2)	88 142 (47.1)	146 356 (55.9)	102 090 (56.4)	469 851 (52.2)
Long-term care	137 314 (50.8)	99 190 (52.9)	115 294 (44.1)	78 788 (43.6)	430 586 (47.8)
Urine cultures per 1000 bed-days, mean (95% CI)^a^					
All	15.1 (11.2-19.0)	11.9 (8.0-15.8)	14.0 (8.6-19.4)	12.6 (10.4-14.8)	13.6 (8.7-18.5)
Acute medical	24.9 (17.8-32.0)	21.1 (15.8-26.4)	20.7 (13.3-28.1)	18.7 (14.1-23.3)	21.6 (14.2-29.0)
Long-term care	5.6 (2.5-8.7)	3.6 (0.6-6.6)	5.5 (2.2-8.8)	4.6 (2.8-6.4)	4.9 (1.5-8.3)
No. of patients with 1 antibiotic prescribed	750	458	712	426	2346
No. of patients with >1 antibiotic prescribed	873	515	754	493	2635
DOT per 1000 bed-days (95% CI)^a^^,^^b^	46.1 (28.8-63.4)	37.0 (22.6-51.4)	46.2 (20.9-71.5)	40.5 (27.4-53.6)	43.1 (22.4-63.8)
LOT per 1000 bed-days (95% CI)^a^^,^^b^	36.7 (23.2-50.2)	29.6 (18.2-41.0)	36.8 (18.3-55.3)	32.8 (21.8-43.8)	34.5 (18.7-50.3)

Abbreviations: DOT, days of antibiotic therapy; LOT, length of antibiotic therapy in days.

^a^ These data are for descriptive purposes only. Comparing the rates before and after the intervention without accounting for any secular trends may result in overestimation or underestimation of the intervention’s effectiveness. The effectiveness of the intervention is estimated by statistical comparison of time trends before and after the intervention that takes account of time trend and autocorrelation among the observations.

^b^ Assessed for antibiotic therapy started temporally in association with a urine culture.

### Interrupted Time Series and DID Analyses

#### Urine Culture Orders

**Figure 2.**
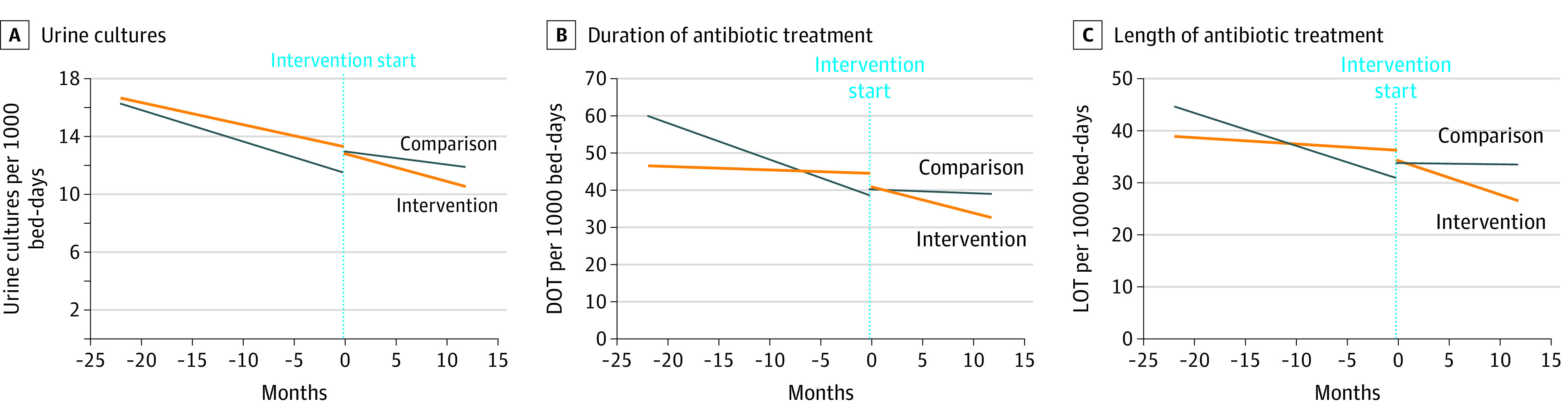
Clinical Outcomes for Urine Cultures, Days of Antibiotic Therapy, and Length of Antibiotic Therapy The clinical outcomes over time for intervention and comparison sites are compared. Slopes of the lines were derived from interrupted time series analysis. Zero on the x-axis marks the start of the intervention. Different sites entered the intervention at different times, so month 1 was not the same calendar month for all sites. All intervention sites’ month 1 data were aggregated into the outcome value for that month; all comparison sites’ month 1 data were aggregated into the outcome value for that month. DOT indicates days of antibiotic therapy; LOT, length of antibiotic therapy in days.

The overall mean number of urine cultures ordered was 15.1 (95% CI, 11.2-19.0) per 1000 bed-days in the baseline period and 11.9 (95% CI, 8.0-15.8) per 1000 bed-days in the intervention period at the intervention sites. The overall mean number of urine cultures was 14.0 (95% CI, 8.6-19.4) per 1000 bed-days in the baseline period and 12.6 (95% CI, 10.4-14.8) per 1000 bed-days in the intervention period at the comparison sites (Table 2). We displayed the time series analysis for urine culture ordering as a graph (Figure 2A) and summarized the results from the segmented regression analysis in Table 3. As shown in Table 3, the coefficient estimates for baseline slopes (β_1_) are negative and significant for both intervention and comparison sites (−0.15 [95% CI, −0.21 to −0.09; *P* = .001] and −0.22 [95% CI, −0.25 to −0.18; *P* = .001], respectively), which indicates that the rate of urine culture ordering decreased in the intervention and comparison sites during the baseline period. The coefficient estimate for the change in regression slope (β_3_) in the intervention sites is not significant (−0.04 [95% CI, −0.17 to 0.09]; *P* = .56), indicating that the rate of urine culture ordering remained unchanged after the intervention (continued to decrease at the same rate). The relative percentage decrease of urine cultures at the intervention sites after the intervention was 6.9%. In contrast, the rate of urine culture ordering increased during the intervention period in the comparison sites, as indicated by significant and positive coefficient estimate (0.13 [95% CI, 0.06-0.19]; *P* = .001) for the change in regression slope during the intervention period compared with the preintervention period (β_3_). The relative percentage increase of urine cultures at the comparison sites in the postintervention period was 28.8%.

**Table 3.** Segmented Regression Analyses for Hypothesis 1: Urine Cultures, DOT, and LOT^a^

Variable	Intervention sites		Comparison sites	
	Coefficient estimate (95% CI)	*P* value	Coefficient estimate (95% CI)	*P* value
**Urine cultures**				
Baseline slope (β_1_)	−0.15 (−0.21 to −0.09)	.001	−0.22 (−0.25 to −0.18)	.001
Change in slope after the intervention (β_3_)	−0.04 (−0.17 to 0.09)	.56	0.13 (0.06 to 0.19)	.001
Postintervention slope	−0.19 (−0.32 to −0.06)	NA	−0.09 (−0.16 to −0.02)	NA
**Urine culture–related DOT**				
Baseline slope (β_1_)	−0.12 (−0.32 to 0.08)	.25	−0.86 (−1.07 to −0.65)	.001
Change in slope after the intervention (β_3_)	−0.54 (−0.93 to −0.15)	.007	0.64 (0.24 to 1.03)	.001
Postintervention slope	−0.66 (−1.11 to −0.28)	NA	−0.22 (−0.53 to 0.32)	NA
**Urine culture–related LOT**				
Baseline slope (β_1_)	−0.12 (−0.28 to 0.05)	.16	−0.62 (−0.78 to −0.46)	.001
Change in slope after the intervention (β_3_)	−0.52 (−0.84 to −0.20)	.001	0.60 (0.30 to 0.90)	.001
Postintervention slope	−0.64 (−0.98 to −0.30)	NA	−0.02 (−0.34 to 0.31)	NA

Abbreviations: DOT, days of antibiotic therapy; LOT, length of antibiotic therapy in days; NA, not applicable.

^a^ A negative coefficient shows a decreasing trend, and a positive coefficient shows an increasing trend. β_3_ estimates the change in trend of outcome during the intervention period compared with the preintervention period.

Because the baseline trends of urine culture ordering were similar for the intervention and comparison sites (decreasing baseline slopes in Table 3), these met the parallel trend assumption required for DID analysis. In the DID analysis for urine culture orders, adjusting for site-specific variability, we found a significant reduction in the number of urine cultures ordered by 3.24 urine cultures per 1000 bed-days (*P* = .003) in the intervention sites compared with comparison sites.

#### Urine Culture–Related DOT

The overall means for urine culture–related DOT were 46.1 (95% CI, 28.8-63.4) in the baseline period and 37.0 (95% CI, 22.6-51.4) in the intervention period at the intervention sites. The overall means for urine culture–related DOT were 46.2 (95% CI, 20.9-71.5) in the baseline period and 40.5 (95% CI, 27.4-53.6) in the intervention period at the comparison sites. The time series analysis for DOT is presented in Figure 2B; the results from the segmented regression analysis are summarized in Table 3. The coefficient estimate for the baseline slope (β_1_) was not significant in the intervention sites (−0.12 [95% CI, −0.32 to 0.08]; *P* = .25), indicating that the rate of urine culture–related DOT remained unchanged during the baseline period (Table 3). In contrast, the coefficient estimate for the baseline slope (β_1_) in the comparison sites was negative (−0.86 [95% CI, −1.07 to −0.65]; *P* = .001), which shows that the rate of DOT was decreasing. The coefficient estimate for the change in regression slope (β_3_) at the intervention sites is significant and negative (−0.54 [95% CI, −0.93 to −0.15]; *P* = .007), which indicates that the rate of urine culture–related DOT decreased after the implementation of the intervention (Table 3). The relative percentage decrease of DOT at the intervention sites after the intervention was 21.7%. In contrast, the coefficient estimate for the change in regression slope (β_3_) at the comparison sites is significant and positive (0.64 [95% CI, 0.24-1.03]; *P* = .001), indicating that the rate of urine culture–related DOT increased in the intervention period at the comparison sites compared with the preintervention period. The relative percentage increase of DOT at the comparison sites in the post-intervention period was 35.1%. We did not perform DID analysis for DOT, because the assumption of parallel trends in the baseline period was not met (baseline slopes in Table 3).

#### Urine Culture–Related LOT

The overall means for urine culture–related LOT were 36.7 (95% CI, 23.2-50.2) in the baseline period and 29.6 (95% CI, 18.2-41.0) in the intervention period at the intervention sites. The overall means for urine culture–related LOT were 36.8 (95% CI, 18.3-55.3) in the baseline period and 32.8 (95% CI, 21.8-43.8) in the intervention period at the comparison sites. We displayed the time series analysis for LOT as a graph (Figure 2C) and summarized the results from the segmented regression analysis in Table 3. The coefficient estimate for the baseline slope (β_1_) was not significant in the intervention sites (−0.12 [95% CI, −0.28 to 0.05]; *P* = .16), indicating that the rate of urine culture–related LOT remained unchanged (Table 3). In contrast, the coefficient estimate for the baseline slope (β_1_) in the comparison sites was negative (−0.62 [95% CI, −0.78 to −0.46]; *P* = .001), which shows that the rate of LOT was decreasing*.* The coefficient estimate for the change in regression slope (β_3_) at the intervention sites was significant and negative (−0.52 [95% CI, −0.84 to −0.20]; *P* = .001), which indicates that the rate of urine culture–related LOT decreased after the implementation of the intervention (Table 3). The relative percentage decrease of DOT at the intervention sites after the intervention was 21.0% (*P* = .001). In contrast, the coefficient estimate for the change in regression slope (β_3_) at the comparison sites was significant and positive (0.60 [95% CI, 0.30-0.90]; *P* = .001), indicating that the rate of urine culture–related LOT increased in the intervention period at the comparison sites compared with the preintervention period. The relative percentage increase of DOT at the comparison sites in the postintervention period was 37.3%. We did not perform DID analysis for LOT, because the assumption of parallel trends in the baseline period was not met (baseline slopes in Table 3).

We repeated the segmented regression analyses without the β_2_ term in the model, thereby excluding the immediate level change after the intervention (eTable 1 in the Supplement). The results were similar to those of the standard segmented regression in Table 3. The site-specific data trends for each of the intervention sites are presented in eTable 2 in the Supplement.

## Discussion

This quality improvement study demonstrates that a novel strategy using external and internal facilitation can be used to effectively implement the evidence-based Kicking CAUTI intervention in both acute and long-term care units across 4 geographically distant sites. The Kicking CAUTI intervention reduced the rate of urine culture ordering and antibiotic use from baseline rates within intervention sites and in contrast to comparison sites. Urine cultures decreased from 15.1 to 11.9 per 1000 bed-days in the intervention sites. This decrease of 3.2 cultures per 1000 bed-days would translate to a reduction of 2881 urine cultures during the 900 437 bed-days of the study. Likewise, the decrease in DOT from 46.1 to 37.0 per 1000 bed-days, or 9.1, would lead to 8193 fewer days of unnecessary antibiotic therapy. LOT decreased from 36.7 to 29.6, or 7.1, which would translate to 6393 fewer days of therapy. Overall, our project led to the desired direction of change (decrease) in all 3 clinical outcomes: urine cultures, DOT, and LOT. More than half of treated patients received more than 1 antibiotic, which underlines the importance of including both DOT and LOT metrics to target both the number of antibiotics used and the true LOT being used.

In contrast, we observed a significant increase in urine cultures, DOT, and LOT in the comparison sites, which is consistent with recent national reports of increased health care–associated infections (including catheter-associated urinary tract infections) during the pandemic.^21^ Patients admitted for COVID-19 infection often receive unnecessary antibiotics and intensive care unit admission,^22,23^ both of which could contribute to the observed increases in urine cultures, DOT, and LOT at the comparison sites. Increasing awareness of antibiotic resistance among uropathogens could also have driven increased antibiotic use. In addition, these increases also may suggest the natural tendency in medicine of “to do more, treat more” if not checked by a focused stewardship program.^24^ Likewise, a prepandemic literature evaluation of antibiotic use in the US did not find a decline over time.^25^

The success of this project implies that our external and internal facilitation^16^ is a viable implementation strategy for implementing stewardship^26^ at a distance. The study team in the coordinating center provided the external facilitation through coaching calls and by troubleshooting logistical challenges. The local champions delivered the interactive, case-based education in small group settings, which encouraged the engagement of frontline clinicians. A modest investment of resources in identifying and training a local internal facilitator may well be worth the significant reduction in infection surveillance and unnecessary antibiotic treatment of ASB. Also, our metrics were derived from a data warehouse, in real time, thus streamlining this project for clinical implementation without medical record reviews.^1^

Comparison of our findings with those of other stewardship interventions for ASB is challenging, because the measurements used across projects vary considerably. Nace et al^27^ decreased antibiotic prescribing for ASB in nursing homes through a multifaceted intervention that included external facilitation (coaching). Chambers et al^28^ conducted a virtual learning collaborative in 45 nursing homes, using diagnostic stewardship, which reduced urine culturing and antibiotic use. On a smaller scale, Doernberg et al^29^ demonstrated the value of having an infectious disease pharmacist make weekly visits to 3 community-based long-term care facilities, leading to a 26% decrease in antibiotic prescriptions for UTI. Acute care stewardship interventions decreased treatment of ASB, one with internal medicine residents^30^ and the other focused on hospitalist physicians.^31^ To our knowledge, the present study is the first to address both acute and long-term care together in the same ASB stewardship intervention in an integrated VA health care system.

### Limitations

Our study has certain limitations, some of which are intrinsic to the use of database measures, others of which are related to the COVID-19 pandemic. Our primary outcome was total urine cultures ordered; this metric does not account for whether the cultures are appropriate or not. Some patients will have legitimate reasons for urine culture. We suspect that many of the urine cultures were inappropriate, extrapolating from the study by Leis et al^32^ in which 68% of urine cultures ordered on inpatients lacked a clinical indication. The differences in our outcomes were relatively small on an absolute basis. When comparing within the intervention sites using statistical comparison of time trends before and after the intervention, we did not find a significant difference in urine cultures before or after the intervention, but the DID analysis comparing intervention with comparison sites showed a significant decrease in urine cultures in the intervention sites after the intervention. Urine culture orders are a blunt measure but are suitable to automation for use outside the scope of a research project. The same limitations apply to DOT and LOT; we know that some antibiotic use in temporal association with urine cultures is appropriate. We excluded antibiotics that were either not relevant to treating UTI or for which therapy was not started in the same time frame as a urine culture. We had hoped to measure sustainability after each site completed a full 12 months in the intervention phase. However, the pandemic placed such time demands on our site champions that we ended data collection in May 2020. Our implementation strategy is likely generalizable to the other VA facilities that have infectious diseases services, an antibiotic stewardship program, and an academic affiliation, but we cannot assess the applicability to health care centers that do not have these characteristics.

## Conclusions

The findings of this quality improvement study suggest that external and internal facilitation can be used to implement the evidence-based Kicking CAUTI intervention. The Kicking CAUTI approach to stewardship resulted in reductions in orders for urine cultures and antibiotic use while being implemented at a distance. In contrast, sites that were not participating in the invention had an increase in urine cultures and antibiotic use temporally associated with urine cultures. Our use of database-derived metrics and a centralized facilitation approach are suitable for further dissemination to affect a greater number of patients and clinicians.

## Notes

Supplement. **eFigure.** The Kicking CAUTI: No Knee-Jerk Antibiotics Campaign Decision-Tree Algorithm **eMethods 1.** Pharmaceutical Agents Excluded From Analysis **eMethods 2.** Details of Segmented Regression of Interrupted Time Series Analysis and Difference-in-Differences Analysis **eTable 1.** Segmented Regression Analyses Without the ß_2_ Term (Immediate Level Change After the Intervention) in the Model **eTable 2.** Site-Specific Data for Monthly Bed-Days and Rates of Urine Cultures and Urine Culture–Related DOT and LOT Rates per 1000 Bed-Days (Intervention Sites)
eReference
