# Supplementary material for: Analysis of an Antibiotic Stewardship Program for Asymptomatic Bacteriuria in the Veterans Affairs Health Care System
Source: JAMA Netw Open. 2022 Jul 25;5(7):e2222530. doi: 10.1001/jamanetworkopen.2022.22530 (PMC9315417; doi:10.1001/jamanetworkopen.2022.22530)
Supplement: Supplement. — eFigure. The Kicking CAUTI: No Knee-Jerk Antibiotics Campaign Decision-Tree Algorithm eMethods 1. Pharmaceutical Agents Excluded From Analysis eMethods 2. Details of Segmented Regression of Interrupted Time Series Analysis and Difference-in-Differences Analysis eTable 1. Segmented Regression Analyses Without the ß2 Term (Immediate Level Change After the Intervention) in the Model eTable 2. Site-Specific Data for Monthly Bed-Days and Rates of Urine Cultures and Urine Culture–Related DOT and LOT Rates per 1000 Bed-Days (Intervention Sites) eReference [file jamanetwopen-e2222530-s001.pdf]

Supplementary Online Content

Grigoryan L, Naik AD, Lichtenberger P, et al. Analysis of an antibiotic stewardship program for asymptomatic bacteriuria in the Veterans Affairs health care system. *JAMA Netw Open*. 2022;5(7):e2222530. doi:10.1001/jamanetworkopen.2022.22530

eFigure. The Kicking CAUTI: No Knee-Jerk Antibiotics Campaign Decision-Tree Algorithm

eMethods 1. Pharmaceutical Agents Excluded From Analysis

eMethods 2. Details of Segmented Regression of Interrupted Time Series Analysis and Difference-in-Differences Analysis

eTable 1. Segmented Regression Analyses Without the $ß_2$ Term (Immediate Level Change After the Intervention) in the Model

eTable 2. Site-Specific Data for Monthly Bed-Days and Rates of Urine Cultures and Urine Culture–Related DOT and LOT Rates per 1000 Bed-Days (Intervention Sites)

eReference

This supplementary material has been provided by the authors to give readers additional information about their work.

© 2022 Grigoryan L et al. *JAMA Network Open*.

eFigure. The Kicking CAUTI: No Knee-Jerk Antibiotics Campaign Decision-Tree Algorithm

This decision aid starts when a provider is assessing a patient who may have a UTI. The front side of the algorithm steps the provider through two questions that should be addressed before the provider sends a urine culture or prescribes antibiotics to treat UTI. We call this process "doing the Texas two-step" to help providers remember that two questions are involved. The back side of the algorithm addresses what to do when the culture results are available, especially if the urine culture is negative, or if the organism that grows proves to be resistant to the antibiotics the patient is receiving. While the emphasis on the front side is on avoiding urine cultures and avoiding the start of antibiotics, the back side helps providers determine when to stop antibiotics that are not necessary.

© 2022 Grigoryan L et al. *JAMA Network Open*.

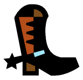

Kicking UTI
The No Knee-Jerk Antibiotics Campaign

Symptomatic UTI versus Asymptomatic Bacteriuria

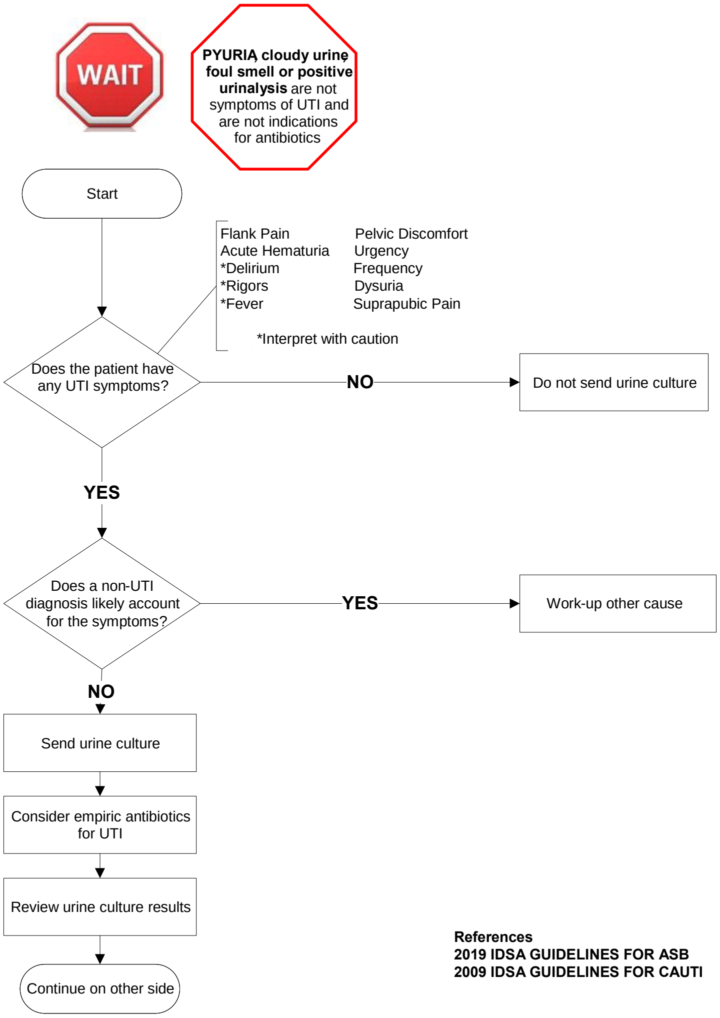

References
2019 IDSA GUIDELINES FOR ASB
2009 IDSA GUIDELINES FOR CAUTI

© 2022 Grigoryan L et al. *JAMA Network Open*.

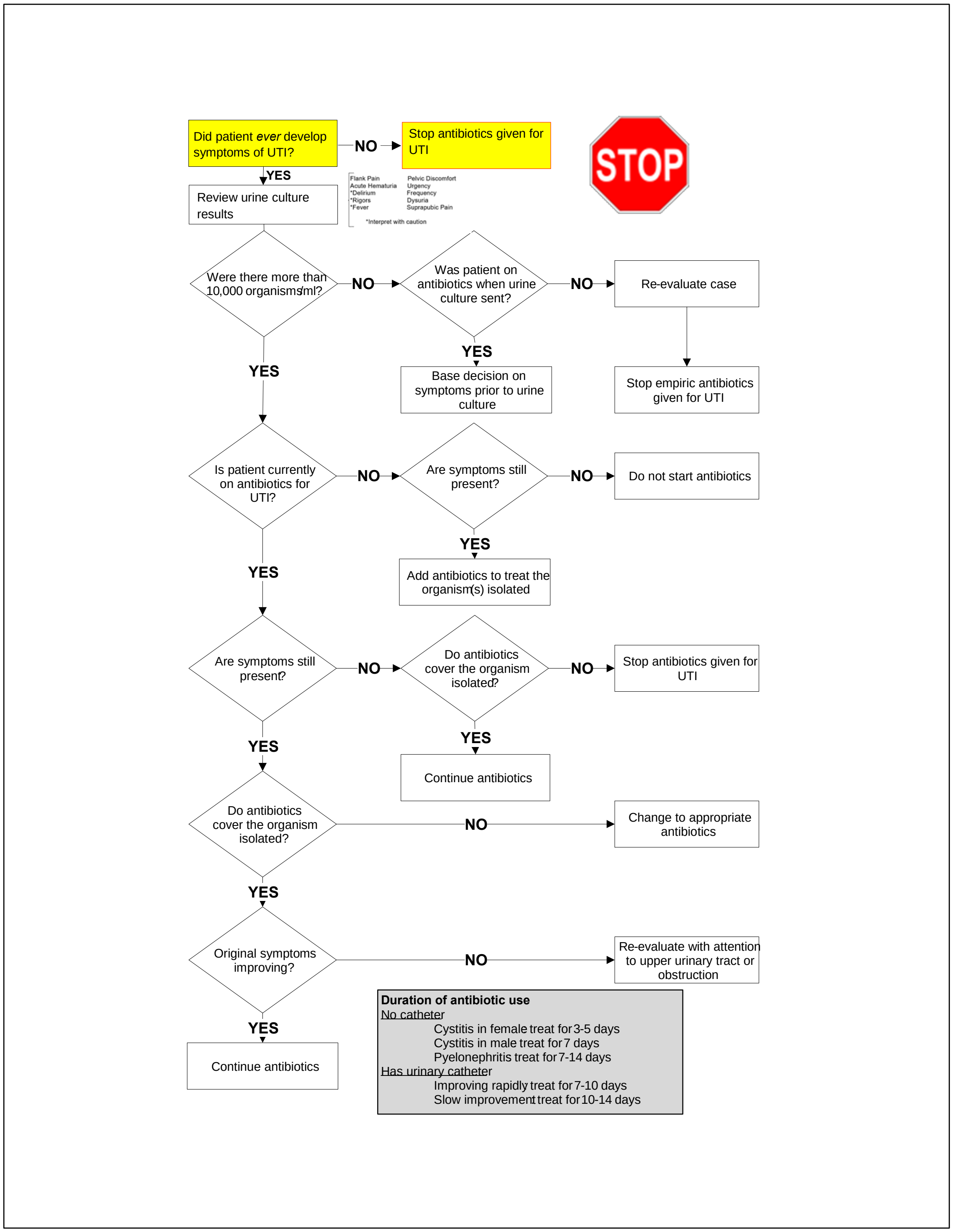

© 2022 Grigoryan L et al. *JAMA Network Open*.

eMethods 1. Pharmaceutical Agents Excluded From Analysis

The following agents are not typically used to treat urinary tract infections and were removed from our pharmacy dataset when calculating antibiotic use: dapsone, clarithromycin, erythromycin, metronidazole, azithromycin, rifampin, rifaximin, rifabutin, clindamycin, dalbavancin, and oral vancomycin.

eMethods 2. Details of Segmented Regression of Interrupted Time Series Analysis and Difference-in-Differences Analysis

Segmented regression: Autoregressive integrative moving average (ARIMA) interrupted time series techniques were used to examine change in slope or level due to the intervention. The following regression model was used: $Rate_t = ß_0 + ß_1 time_t + ß_2 intervention_t + ß_3 time\_after\_intervention_t + e_t$., where $Rate_t$ is the outcome for each model (urine cultures, DOT and LOT per 1,000 bed-days). The regression parameters were: $ß_0$ (baseline **level** of the outcome); $ß_1$ (baseline **trend**, that is, the change in outcome per month in the baseline segment, $ß_2$ (the change in **level** of outcome in the post-intervention segment); $ß_3$ (the change in **trend** of outcome in the post-intervention segment); e_t estimates the error.

We conducted separate segmented regression analyses for each outcome for the intervention and comparison sites separately. We expected that our intervention would have a gradual effect, therefore, we focused our results on the interpretation of $ß_3$ (the change in **trend** of outcome in the post-intervention segment) instead of the immediate level change ($ß_2$) following the intervention ($ß_2$) as recommended by the ITS tutorial.[1] We adjusted the time series regression for auto-correlation and moving averages to mitigate large fluctuations in the data.

© 2022 Grigoryan L et al. *JAMA Network Open*.

Difference-in-differences (DID) analysis: In the DID approach, we used multiple linear mixed-model regression analysis to study the impact of the intervention on each outcome (urine cultures, urine-culture related DOT and LOT per 1,000 bed-days). We added month as a predictor variable and added an interaction term between study site (intervention and comparison) and study period (baseline and intervention) to test the hypothesis that urine culture ordering would decrease at the intervention sites and would remain unchanged at the comparison sites. The model included a random site effect to account for correlation between the observations at the same site.

© 2022 Grigoryan L et al. *JAMA Network Open*.

eResults

eTable 1. Segmented Regression Analyses Without the ß$_2$ Term (Immediate Level Change After the Intervention) in the Model

Segmented regression analyses for Hypothesis 1: urine cultures, DOT, and LOT						
	**Intervention sites**			**Comparison sites**		
	Coefficient estimate	95% CI	P value	Coefficient estimate	95% CI	P value
Urine-cultures						
Baseline slope (ß$_1$)	**-0.15**	**(-0.21; -0.09)**	**0.001**	**-0.22**	**(-0.26; -0.19)**	**0.001**
Change in slope after the intervention (ß$_3$)	-0.04	(-0.08; 0.002)	0.07	**0.08**	**(0.04; 0.12)**	**0.001**
Post-intervention slope	**-0.19**	**(-0.21; -0.16)**		**-0.15**	**(-0.10; -0.08)**	
Urine-culture related days of therapy (DOT)						
Baseline slope (ß$_1$)	-0.08	(-0.29; 0.13)	0.44	**-0.92**	**(-1.16; -0.68)**	**0.001**
Change in slope after the intervention (ß$_3$)	**-0.29**	**(-0.43; -0.14)**	**0.001**	**0.29**	**(0.13; 0.46)**	**0.001**
Post-intervention slope	**-0.37**	**(-0.78; -0.61)**		**-0.63**	**(-0.20; -0.01)**	
Urine-culture related length of therapy (LOT)						
Baseline slope (ß$_1$)	-0.08	(-0.27; 0.12)	0.44	**-0.69**	**(-0.89; -0.48)**	**0.001**
Change in slope after the intervention (ß$_3$)	**-0.22**	**(-0.35; -0.08)**	**0.003**	**0.23**	**(0.09; 0.38)**	**0.002**
Post-intervention slope	**-0.29**	**(-0.72; -0.56)**		-0.46	(-0.10; 0.06)	
CI=confidence intervals; Significant results are bolded. A negative coefficient shows a decreasing trend, and a positive coefficient shows an increasing trend. ß$_3$ estimates the **change in trend** of outcome in the post-intervention segment.						

© 2022 Grigoryan L et al. *JAMA Network Open*.

eTable 2. Site-Specific Data for Monthly Bed-Days and Rates of Urine Cultures and Urine Culture–Related DOT and LOT Rates per 1000 Bed-Days (Intervention Sites)

Months from	Total Bed-days				
intervention	Intervention Site 1	Intervention Site 2	Intervention Site 3	Intervention Site 4	Aggregated
-20	2664				2664
-19	2620				2620
-18	2653		5233		7886
-17	2652		4954		7606
-16	2366		5163	3829	11358
-15	2667	3906	5342	3662	15577
-14	2587	3505	4855	3701	14648
-13	2642	3454	5036	3899	15031
-12	2665	3701	4634	3772	14772
-11	2576	3306	4572	3896	14350
-10	2628	3735	4310	3798	14471
-9	2718	3699	4585	3889	14891
-8	2661	3715	4921	3730	15027
-7	2447	3422	5073	3932	14874
-6	2638	3785	4915	3856	15194
-5	2720	3674	4670	3848	14912
-4	2286	3678	5222	3907	15093
-3	2561	3658	5312	3753	15284
-2	2553	3356	4697	3889	14495
-1	2625	3427	5417	3803	15272
0	2435	3612	4993	3512	14552
1	2252	3316	5136	3846	14550
2	2140	3772	4893	3789	14594
3	2498	3626	5037	3931	15092
4	2740	3634	5095	3812	15281
5	2541	3605	5180	3933	15259
6	2613	3769	5294	3845	15521
7	2867	3754	5330	3704	15655
8	2818	3811	5421	3798	15848
9	2090	3729	5528	3755	15102
10	647	3343	5109	3942	13041
11		3447	5137	4022	12606
12		3498	4198	3541	11237
13		3420		3044	6464
14		3176		1107	4283
15		2799			2799

© 2022 Grigoryan L et al. *JAMA Network Open*.

Months from	Urine Cultures-Standardized by 1000 bed-days				
intervention	Intervention Site 1	Intervention Site 2	Intervention Site 3	Intervention Site 4	Aggregated
-20	13.889				13.889
-19	15.267				15.267
-18	15.454		16.434		16.104
-17	15.460		11.304		12.753
-16	14.793		13.558	31.601	19.898
-15	17.998	13.569	13.104	25.669	17.012
-14	12.370	12.268	11.123	27.020	15.634
-13	15.140	14.186	15.488	21.288	16.632
-12	13.884	19.995	13.164	23.595	17.669
-11	12.034	19.056	10.936	18.737	15.122
-10	11.035	12.316	10.905	19.747	13.613
-9	14.717	14.058	11.559	16.457	14.035
-8	9.395	11.036	14.022	29.491	16.304
-7	8.991	16.657	11.433	22.380	15.127
-6	13.647	19.551	12.411	20.228	16.388
-5	17.279	14.426	8.994	15.333	13.479
-4	16.623	14.138	17.235	16.893	16.299
-3	14.838	14.489	13.178	15.721	14.394
-2	12.143	14.899	8.516	14.142	12.142
-1	10.286	12.839	7.938	16.566	11.590
0	15.606	14.120	10.815	14.237	13.263
1	15.542	14.777	10.125	15.081	13.333
2	11.682	16.967	14.511	15.835	15.075
3	10.008	9.377	9.927	11.193	10.138
4	13.504	9.906	13.739	11.542	12.237
5	13.381	7.212	11.776	10.170	10.551
6	20.666	10.878	12.089	12.484	13.337
7	10.464	12.786	12.383	14.849	12.712
8	9.936	9.184	11.806	15.534	11.736
9	12.919	9.654	9.045	14.115	10.992
10	23.184	9.871	13.506	14.713	13.419
11		6.963	7.981	12.183	9.043
12		7.147	10.481	14.403	10.679
13		12.573		12.484	12.531
14		5.668		14.453	7.938
15		8.932			8.932

© 2022 Grigoryan L et al. *JAMA Network Open*.

Months from	Urine-culture related DOT per 1,000 bed-days				
intervention	Intervention Site 1	Intervention Site 2	Intervention Site 3	Intervention Site 4	Aggregated
-20	43.544				43.544
-19	48.473				48.473
-18	49.378		41.277		44.002
-17	33.183		18.975		23.928
-16	53.677		41.449	83.050	58.021
-15	64.117	52.227	44.927	83.561	59.126
-14	44.840	28.531	43.666	78.087	48.949
-13	55.640	50.376	37.728	41.549	44.774
-12	42.402	68.630	41.217	57.264	52.396
-11	61.724	60.496	35.652	61.602	53.101
-10	36.530	43.909	23.898	47.920	37.662
-9	46.726	33.793	22.246	45.770	35.726
-8	30.440	36.608	23.369	82.842	42.657
-7	26.563	38.866	23.852	89.522	45.112
-6	54.587	58.917	23.601	57.573	46.400
-5	70.588	39.194	25.268	42.620	41.443
-4	75.678	41.599	51.130	77.297	59.299
-3	62.085	46.473	45.745	45.031	48.482
-2	32.511	41.418	18.310	66.598	39.117
-1	43.810	37.642	17.722	45.227	33.525
0	61.191	55.925	55.478	40.718	52.982
1	61.279	47.648	22.002	48.622	40.962
2	27.103	87.222	29.021	48.034	48.719
3	41.233	35.025	23.427	26.456	29.950
4	56.204	25.592	44.750	46.957	42.798
5	44.864	20.527	24.131	27.206	27.525
6	72.331	22.022	28.712	38.752	36.918
7	63.481	60.202	35.272	40.497	47.653
8	45.422	21.779	33.942	49.763	36.850
9	37.799	18.504	24.964	46.338	30.460
10	72.643	19.743	45.606	57.331	43.862
11		23.209	21.803	35.306	26.495
12		20.011	32.873	54.222	35.597
13		37.719		26.610	32.488
14		25.819		30.714	27.084
15		34.655			34.655

© 2022 Grigoryan L et al. *JAMA Network Open*.

Months from	Urine-culture related LOT by 1000 bed-days				
intervention	Intervention Site 1	Intervention Site 2	Intervention Site 3	Intervention Site 4	Aggregated
-20	33.033				33.033
-19	38.168				38.168
-18	35.809		37.837		37.154
-17	26.018		17.763		20.642
-16	43.533		35.832	64.508	47.103
-15	46.119	38.146	35.380	66.357	45.195
-14	34.789	23.680	36.045	61.875	39.391
-13	47.313	40.243	28.793	31.034	35.260
-12	33.396	48.906	36.470	51.697	42.919
-11	43.478	50.514	30.402	44.918	41.324
-10	35.388	32.396	20.650	37.915	30.889
-9	35.320	26.764	17.666	31.885	26.862
-8	25.554	27.725	19.102	64.879	33.739
-7	22.477	33.314	20.304	63.835	35.162
-6	42.456	47.556	22.177	39.938	36.528
-5	56.250	28.851	20.128	37.162	33.262
-4	59.055	30.723	39.640	60.660	45.849
-3	42.952	36.085	37.462	35.172	37.490
-2	29.377	37.545	16.606	53.227	33.529
-1	28.571	27.721	14.399	38.917	25.930
0	50.103	40.975	49.870	36.162	44.393
1	53.286	31.363	19.665	38.482	32.509
2	21.028	66.013	26.364	37.477	38.715
3	36.029	26.200	19.456	20.860	24.185
4	45.985	20.638	37.684	34.627	34.356
5	38.174	16.089	21.429	22.375	23.199
6	55.874	18.307	20.778	31.990	28.864
7	46.041	47.949	28.330	31.587	37.049
8	36.906	16.269	30.622	42.391	31.108
9	32.536	14.749	20.441	37.284	24.897
10	61.824	15.555	34.840	44.394	34.123
11		17.987	18.883	26.604	21.101
12		17.724	27.632	44.055	29.723
13		27.193		21.025	24.288
14		18.262		26.197	20.313
15		26.438			26.438

© 2022 Grigoryan L et al. *JAMA Network Open*.

eReference

1. Bernal JL, Cummins S, Gasparrini A. Interrupted time series regression for the evaluation of public health interventions: a tutorial. Int J Epidemiol. Feb 1 2017;46(1):348-355. doi:10.1093/ije/dyw098

© 2022 Grigoryan L et al. *JAMA Network Open*.
